# Immune dysregulation in the prostates of C57BL/6 ^*Aire*−/−^ mice mirrors that seen in human benign prostatic hyperplasia

**DOI:** 10.1002/path.70093

**Published:** 2026-07-08

**Authors:** Nadia A Lanman, Meaghan M Broman, Harish Kothandaraman, Gregory M Cresswell, Gada D Awdalkreem, Dilinaer Wusiman, Andree K Kolliegbo, Alexander P Glaser, Brian T Helfand, Renee E Vickman, Jiang Yang, Simon W Hayward, Timothy L Ratliff

**Affiliations:** ^1^ Department of Comparative Pathobiology Purdue University West Lafayette IN USA; ^2^ Purdue University Institute for Cancer Research Purdue University West Lafayette IN USA; ^3^ Department of Computer Science Purdue University West Lafayette IN USA; ^4^ Division of Urology, Department of Surgery Endeavor Health Research Institute Evanston IL USA; ^5^ University of Chicago Pritzker School of Medicine Chicago IL USA; ^6^ Department of Cancer Biology Endeavor Health Research Institute Evanston IL USA

**Keywords:** inflammation, scRNA‐seq, *Aire*, prostate, BPH, aging, inflammaging

## Abstract

Benign prostatic hyperplasia (BPH) is the most common urologic disease in aging men, resulting in significant morbidity. The etiologies of BPH are unknown, though chronic prostatic inflammation is known to promote hyperplasia, fibrotic remodeling, and therapeutic resistance in BPH. BPH is highly complex and heterogeneous, presenting with varying degrees of stromal and epithelial proliferation, fibrosis, inflammation, and associated lower urinary tract symptoms. This complexity poses challenges in developing models. Here, we characterize an *Aire* transcription factor‐deficient nonresolving (chronic) inflammation model in a C57BL/6J background for the study of the prostatic inflammation present in BPH. This chronic inflammatory model exhibits a lack of central tolerance but retains an otherwise intact and functional immune system. C57BL/6^*Aire*−/−^ mice were subcutaneously injected with prostate homogenate protein and Freund's Complete Adjuvant and boosted after 10 days. After 21 and 35 days, whole prostates were collected for histology, flow cytometry, and single‐cell RNA sequencing (scRNA‐seq), which were then compared to human BPH data. Inflammation was confined to the prostate in C57BL/6^*Aire*−/−^ mice. Histological and scRNA‐seq data show that the dominant leukocyte phenotypes in the prostates of C57BL/6^*Aire*−/−^ mice are B and T lymphocytes. Macrophages in C57BL/6^*Aire*−/−^ mouse prostates express signatures associated with an array of phenotypes, as also seen in BPH. We identify a *Trem2* ^+^ population of macrophages and aging‐associated Cd8^+^ *GZMK* hi *GZMB* low T cells in C57BL/6^*Aire*−/−^ prostates similar to those seen in human BPH. Further, fibroblast clusters in C57BL/6^*Aire*−/−^ are similar to fibroblasts identified from the prostates of patients diagnosed with BPH, and these clusters also express markers associated with aging. Overall, the inflammation and predicted leukocyte‐‐stromal interactions observed in the prostates of C57BL/6^*Aire*−/−^ mice resemble human BPH, likely at the early stages of the disease, making this model useful for studying the impact of inflammation‐driven prostatic hyperplasia. © 2026 The Author(s). *The Journal of Pathology* published by John Wiley & Sons Ltd on behalf of The Pathological Society of Great Britain and Ireland.

## Introduction

Benign prostatic hyperplasia (BPH) affects nearly 75% of men aged 60–69 years and over 80% of men 70 years and older [1]. This chronic, progressive condition involves nonmalignant proliferation of epithelial and stromal cells, causing lower urinary tract symptoms like frequency, urgency, nocturia, and incomplete voiding [2]. Despite its prevalence, the underlying causes of BPH remain unknown, contributing to a 45% treatment failure rate due to incomplete understanding of its pathogenesis and a focus on prostate physiology rather than disease mechanisms [3].

BPH involves complex interactions between hormonal factors [4], epithelial‐stromal interactions, genetic factors, and inflammatory pathways, causing cell growth in the periurethral transition zone [5, 6, 7, 8, 9, 10, 11]. While the precise role of inflammation in prostatic hyperplasia is unclear, studies have found that nearly all BPH prostates have inflammatory infiltrates and that around 75% of men with BPH show evidence of significant prostate inflammation on histological examination [12, 13, 14]. Recent studies also suggest that inflammatory signaling dysregulation promotes leukocyte accumulation and hyperplasia [9, 10, 15, 16, 17]. TNF release from macrophages encourages fibroblast proliferation, while TNF blockade reduces prostatic hyperplasia in mouse models and humans and is associated with lower BPH incidence and reduced prostate size [10, 18].

Several rodent models have been used to study prostatic inflammation and hyperplasia, including diet‐ and hormone‐inducible systems [19, 20, 21, 22, 23]. The testosterone plus estradiol (T + E2) hormone recapitulates the hormonal environment of aging men but lacks immune specificity [21, 24, 25]. The prolactin‐overexpressing probastin (Pb)‐PRL transgenic mouse develops prostatic hyperplasia and inflammation but shows reduced androgen receptor expression and resistance to 5α‐reductase inhibitors (5‐ARIs) [22, 23, 26, 27]. Nonobese diabetic (NOD) mice exhibit organ‐specific inflammation, including in the prostate, but their Th1‐biased immunity limits broader applicability [28, 29, 30, 31]. The Prostate Ovalbumin Expressing Transgenic‐3 (POET‐3) model provides antigenic specificity and demonstrates inflammation‐driven hyperplasia, though its responses are transient [20, 32, 33, 34, 35]. Each model thus has distinct limitations, underscoring the need for better systems that capture the chronic inflammation and hyperplasia characteristic of human BPH [10, 17].

We previously showed that autoimmune disease increased BPH risk and that anti‐inflammatory therapies could suppress BPH [10]. We therefore evaluated a novel use of the *Autoimmune Regulator* (*Aire*) KO mouse model (C57BL/6^*Aire*−/−^) for studying the impact of inflammation on prostate and hyperplasia mechanisms. *Aire* prevents autoimmunity by inducing deletion of high‐affinity T cells in thymic epithelial cells [36, 37]. *Aire* deficiencies cause loss of central tolerance and autoimmunity development [36, 37, 38]. The C57BL/6^*Aire*−/−^ model, which is induced by prostate homogenate immunization [28, 29, 39], is a benign model exhibiting chronic, nonresolving inflammation localized only to the prostate, preserving normal systemic immune responses [40]. This model could be valuable for studying chronic prostatic inflammation in BPH. Here, we characterize the C57BL/6^*Aire*−/−^ mouse, study the effects of inflammation in the prostate, and compare this model with WT C57BL/6J mice and human BPH tissue.

## Materials and methods

### Ethics approval

All human tissue procurement was performed in accordance with protocols approved by the Endeavor Health (formerly NorthShore University HealthSystem) Institutional Review Board.

All mouse procedures were performed in accordance with Purdue Animal Care and Use Committee‐approved protocols.

### Mice

Male C57BL/6^*Aire*−/−^ mice 8–12 weeks old were subcutaneously injected with 200 μg of prostate homogenate protein from all four lobes of the prostate and Freund's Complete Adjuvant (Thermo Fisher Scientific, Waltham, MA, USA) in a 1:1 ratio for a total volume of 100 μl. After 10 days, mice were boosted subcutaneously with 200 μg prostate homogenate protein and Freund's Incomplete Adjuvant (Thermo Fisher Scientific) at a 1:1 ratio for a total volume of 100 μl. All four lobes of the prostates were collected at 21 days, which represents a reasonable time point for assessing overall inflammation based on prior work [39], or 35 days after booster, which was included as a subsequent time point to account for potential fluctuations in leukocyte populations. One of each paired lobe of every prostate was fixed in 10% neutral buffered formalin (NBF) for histology, and the other was processed for single‐cell RNA sequencing (scRNA‐seq).

### Mouse necropsy and histology

Samples of mouse prostate as well as lung, kidney, liver, spleen, and pancreas were collected into 10% NBF, embedded in paraffin, and sectioned and stained with H&E. For prostate immunofluorescence (IF) or immunohistochemical (IHC) staining (antibodies listed in supplementary material, Table S1), formalin‐fixed paraffin‐embedded (FFPE) sections were deparaffinized, and antigen retrieval was performed at 95 °C for 20 min in a BioCare decloaking chamber (BioCare Medical, Pacheco, CA, USA).

### Human prostate specimens

Human prostatic tissues were obtained as previously described [10, 17].

### Cell flow cytometry

All four lobes of mouse prostates were collected from C57BL/6^*Aire*−/−^ mice, digested, and processed to a single‐cell suspension, followed by cell flow cytometry (supplementary material, Table S1), as previously described [33, 41].

### Single‐cell RNA sequencing

Six male C57BL/6^*Aire*−/−^ mice were used in scRNA‐seq experiments, with prostates pooled from three mice for each scRNA‐seq sample. Half of all four prostate lobes of prostates were digested in complete RPMI with 1 mg/ml Collagenase I on a shaker at 225 rpm and 37 °C for 1.5 h. Cells were prepped with the Chromium Next GEM Single Cell 3’ Kit version 3.1 (10× Genomics, Pleasanton, CA, USA) following the manufacturer's protocol. Matrices were analyzed using Seurat version 4.1.0 R [42, 43].

### Statistical analyses

Wilcoxon rank‐sum tests identified marker genes and macrophage polarization differences. Negative binomial tests identified differential gene expression between WT and C57BL/6^*Aire*−/−^ mice. Benjamini–Hochberg‐adjusted *p* values were used.

## Results

### Immunized C57BL/6^*Aire*^ ^−/−^ mice consistently develop persistent and localized prostatic inflammation

**Figure 1:**
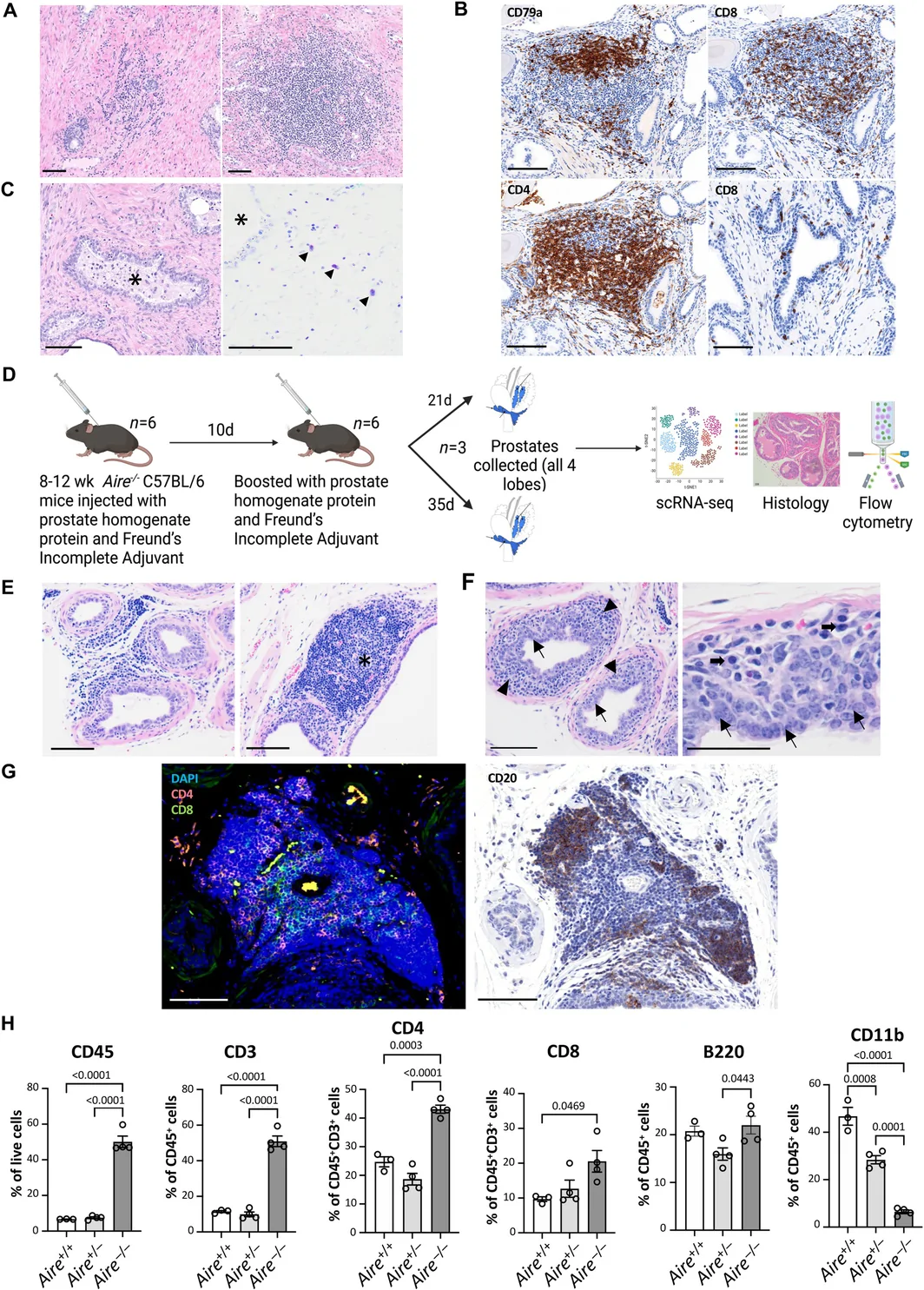
Cellular composition and architecture of C57BL/6^*Aire−*/−^ prostates and comparison with human prostates. (A) H&E sections of human prostate immune cells showing stromal lymphoid cells in loose aggregates (left) to dense lymphoid structures (right). (B) IHC of stromal lymphocytes in organizing lymphoid structures, highlighting CD79a^+^ B cells (top left), CD8^+^ T cells (top right), CD4^+^ T cells (bottom left), and intraepithelial CD8^+^ T cells (arrowheads). (C) H&E section (left) of human prostate showing intraluminal macrophages (asterisk). Toluidine blue‐stained section (right) highlighting stromal mast cells (arrowheads) adjacent to gland (asterisk). (D) Overview of mouse experimental design. *Aire* KO mice in a C57BL/6 background were injected with prostate homogenate protein and Freund's Incomplete Adjuvant. After 10 days, mice again received a booster of prostate homogenate protein and Freund's Incomplete Adjuvant. All four lobes of the prostates were harvested at either 21 days or at 35 days after receiving the booster and were subjected to scRNA‐seq, histological analysis, or cell flow cytometry. Created in BioRender. Lanman, N. (2026) https://BioRender.com/xk0z12a. (E) H&E sections of dorsal prostate from immunized C57BL/6^*Aire*−/−^ mouse prostates with prominent lymphoid aggregates (left) and organizing lymphoid structures (right) with germinal center‐like areas (asterisks). (F) Dorsal prostate of immunized C57BL/6^*Aire*−/−^ mouse showing infiltration of lymphoid cells (arrowheads) adjacent to glandular epithelium (arrows). Infiltrating cells consist of lymphoid cells including numerous plasma cells (bold arrows). (G) IF (left) and IHC (right) highlighting arrangement of CD4 (orange) and CD8 (green) T cells and CD20 (DAB) B cells in stromal lymphoid aggregates. (H) Immune cell flow cytometry of Day 31 post‐immunization C57BL/6^*Aire*−/−^, C57BL/6^*Aire*+/−^, and C57BL/6^*Aire*+/+^ mouse prostates. Scale bars, 100 μm. Flow cytometry data are presented as mean ± SEM.

Inflammatory infiltrates were detected in all human BPH specimens included in this study, as demonstrated by scRNA‐seq and histological evaluation. Histological analyses also indicated that T cells were the dominant immune cell subtype, followed by macrophages [15]. Histologically, most immune cells were present in the stroma of human BPH prostates (Figure 1A). Stromal lymphoid cells were arranged in scattered individual cells, periglandular or perivascular loose aggregates, or organizing follicle‐like structures, occasionally forming germinal center‐like areas (Figure 1A,B). Aggregates and organizing lymphoid structures were composed of CD4^+^ T cells mixed with CD8^+^ T cells and CD79a^+^ B cells (Figure 1B). Intraepithelial lymphocytes were predominantly CD8^+^ T cells (Figure 1B). Myeloid cells, predominantly macrophages and mast cells, were observed within the glandular lumina and scattered within the stroma (Figure 1C).

Histological analysis, scRNA‐seq, and cell flow cytometry were performed on C57BL/6^*Aire*+/+^, C57BL/6^*Aire*+/−^, and C57BL/6^*Aire*−/−^ mouse prostates (Figure 1D). In C57BL/6^*Aire*+/+^ and C57BL/6^*Aire*+/−^ mice, prostatic immune cell populations were minimal (supplementary material, Figure S1). In contrast, C57BL/6^*Aire*−/−^ mice consistently developed prostatic inflammation, which persisted over time (Figure 1E,F). Immune cell infiltrates were observed most often within the anterior and dorsal prostate lobes, but lobes were variably affected (supplementary material, Figures S1 and S2). Like human BPH prostates, immune infiltrates were most notably present within the stroma (Figure 1E,F). These infiltrates were composed predominantly of lymphoid cells, which were variably arranged in perivascular and periglandular aggregates to organize lymphoid structures, sometimes forming one or more germinal centers (Figure 1E,F). Lymphoid aggregates were composed of CD4^+^ T cells, CD8^+^ T cells, and CD20^+^ B cells (Figure 1G; supplementary material, Figure S1). In the epithelium, plasma cells and lymphocytes often infiltrated the mucosal lamina propria (Figure 1F). As expected in an autoimmune model, CD11b^+^ myeloid cells were present in relatively low numbers, as were mast cells (Figure 1H; supplementary material, Figure S1) [44, 45]. Furthermore, histologically significant inflammation was not detected in organs outside the prostate (lung, liver, kidney, pancreas) or in local tissues such as the seminal vesicles (supplementary material, Figure S3), even in C57BL/6^*Aire*−/−^ mice, consistent with previous descriptions of a relatively mild *Aire* ^*−/−*^ phenotype in the C57BL/6 background [36, 46].

**Figure 2:**
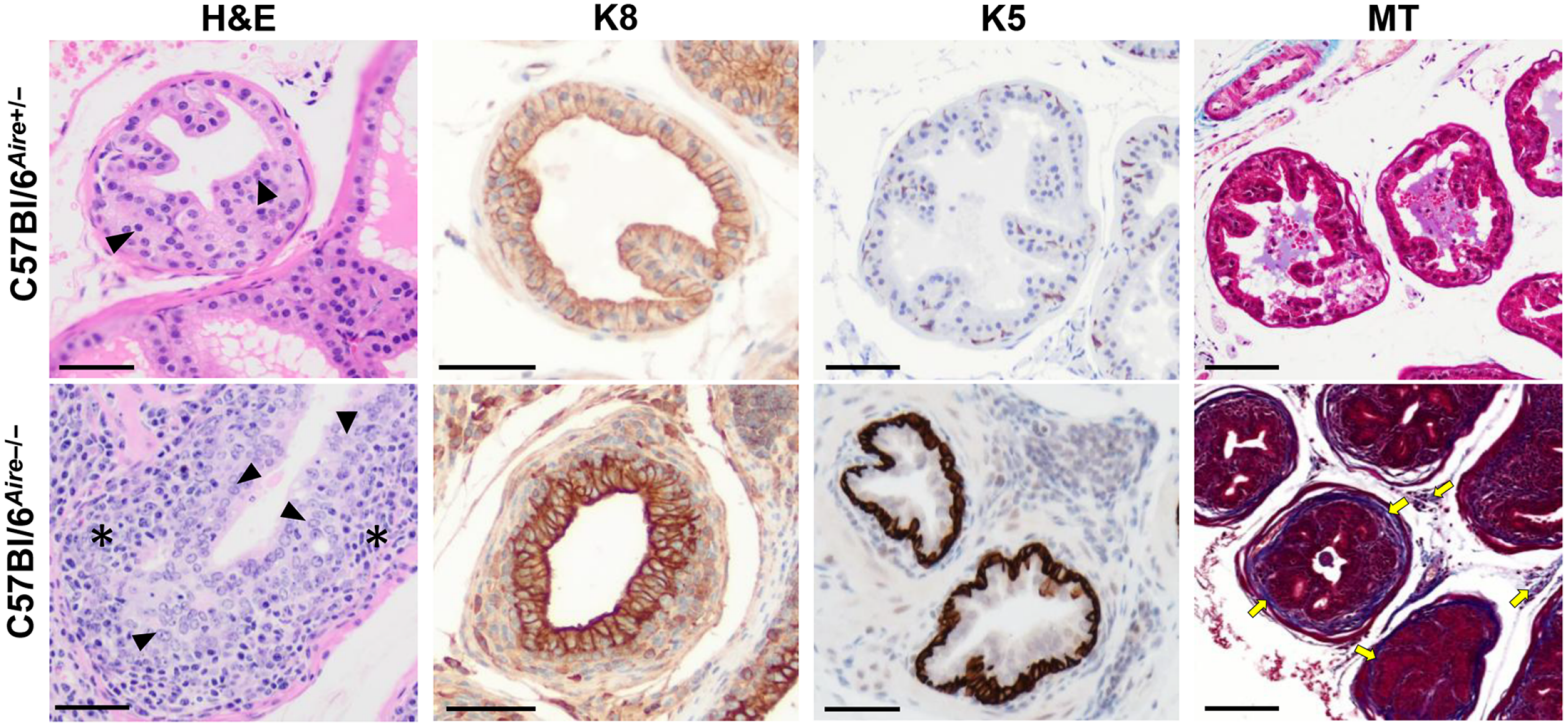
Epithelial and stromal expansion in C57BL/6^*Aire*−/−^ mouse prostates. Representative images of dorsal lobes of 3‐ to 4‐month‐old C57BL/6^*Aire*+/−^ (top) and C57BL/6^*Aire*−/−^(bottom) mouse prostates in H&E, K8 luminal epithelial cell IHC, K5 basal epithelial cell IHC, and Masson's trichrome (MT) stained sections. Note the epithelial immune cell infiltration (asterisks) and epithelial cell expansion (arrowheads) in C57BL/6^*Aire*−/−^ prostates (far left). Epithelial expansion includes both K8^+^ luminal (middle left) and K5^+^ basal (middle right) cells. Note blue‐staining stromal collagen expansion (yellow arrows) in C57BL/6^*Aire*−/−^ prostates (far right). Scale bars, 100 μm.

Consistent with histological findings, flow cytometric analyses of the prostate immune cell populations showed an overall predominance of lymphoid cells, with CD3^+^ T cells comprising the largest proportion of CD45^+^ leukocytes followed by B220^+^ B cells and a relatively low proportion of CD11b^+^ monocyte/macrophage populations (Figure 1H). Additionally, expansion of both epithelial and stromal cell components was also observed in C57BL/6^*Aire*−/−^ mice (Figure 2), consistent with studies demonstrating inflammation‐induced epithelial and stromal hyperplasia in BPH. Epithelial expansion included both keratin 8 (K8^+^) luminal and keratin 5 (K5^+^) basal epithelial cells (Figure 2). Additionally, C57BL/6^*Aire*−/−^ prostates showed clear evidence of collagen deposition, consistent with ongoing stromal remodeling (Figure 2). This cellular and stromal expansion did not translate into a significant difference in voiding symptoms as demonstrated by the void spot assay (VSA) (supplementary material, Figure S4).

Collectively, these results confirm the presence of chronic prostate‐confined inflammation within C57BL/6^*Aire*−/−^ mice but not C57BL/6^*Aire*+/+^ and C57BL/6^*Aire*+/−^ mice. The immune cell composition of C57BL/6^*Aire*−/−^ mouse prostates and human BPH prostates demonstrates several similarities. Additionally, the immune cell composition and morphology in both species, particularly the presence of organizing lymphoid structures, is suggestive of chronic antigenic stimulation and a robust localized adaptive immune response. However, while evidence of inflammation and cellular and stromal expansion similar to that of human BPH prostates is observed, evidence of a significant impact on urinary tract function was not observed, highlighting a notable difference in the clinical presentation between the C57BL/6^*Aire*−/−^ model and human BPH.

### B and T lymphocytes are the dominant immune phenotypes in C57BL/6^*Aire*^ ^−/−^ mouse prostates

**Figure 3:**
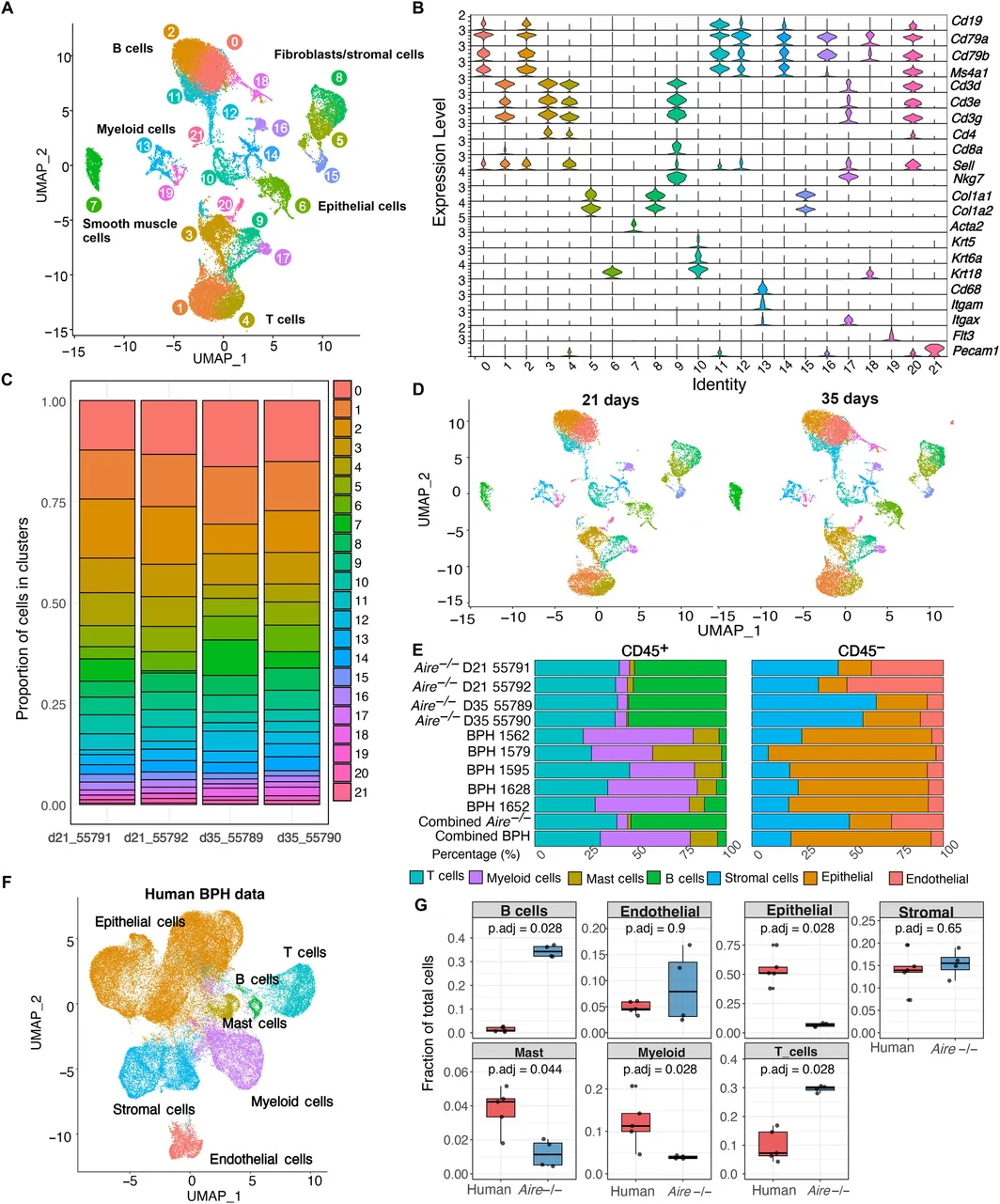
S‐cRNA‐seq of C57BL/6^*Aire*−/−^ mouse prostates. (A) Combined clustering of all cells. A total of 22 clusters were detected and assigned to one of six major cell types. (B) Stacked violin plot of canonical cell markers used to assign cell types to clusters. (C) Stacked bar chart depicts proportions of cells from each cluster across all *Aire* ^−/−^ samples that were sequenced. (D) UMAP plot split to separate samples harvested 21 days after receiving a booster or 35 days after receiving a booster. (E) Horizontal stacked bar plot depicting cellular composition of each C57BL/6^*Aire*−/−^ mouse sample as well as five human samples derived from BPH patients [10, 17]. The bottom two bars show the cellular composition of combined C57BL/6^*Aire*−/−^ or human samples. (F) UMAP of scRNA‐seq data from human BPH tissue adapted from other works [10, 17]. (G) Cellularity boxplots depicting cellular composition in mouse and human, along with adjusted *p* values highlighting statistically significant compositional differences between mouse and human.

To gain further insight into the populations of cells present in all lobes of C57BL/6^*Aire*−/−^ mouse prostates, scRNA‐seq was performed. A total of 14,935 cells were sequenced from pooled prostates of three C57BL/6^*Aire*−/−^ mice harvested at 21 days after the booster, and 17,149 cells were sequenced from three pooled C57BL/6^*Aire*−/−^ mouse prostates harvested at 35 days after the booster (supplementary material, Table S2). The median unique molecular identifiers detected in each cell was 3,048, and the median number of genes detected per cell was 1,361. Initially, 22 cell clusters were defined (Figure 3A; supplementary material, Table S3). Cells in each cluster were classified into various immune and nonimmune cell types using canonical marker expression (Figure 3B; supplementary material, Table S4) as well as automatic cell type identification using SingleR [47] (supplementary material, Figure S5A). Immune cells were abundant in the scRNA‐seq data, with lymphoid cells being the dominant phenotype. In line with the paucity observed in histology, granulocytes were absent in the scRNA‐seq data. The predominance of lymphocytes over granulocytes is consistent with an autoimmune‐driven inflammatory response rather than one triggered by infection [44, 45]. Clusters 0, 2, 11, 12, 14, and 20 were B‐cell clusters (*Cd19* ^+^ *Ms4a1* ^+^ *Cd79a* ^+^ *Cd79b* ^+^) that comprise 38% of the cells. Overall, 32% of the cells were T cells (*Cd3d* ^+^ *Cd3e* ^+^ *Cd3g* ^+^), composed of clusters 1, 3, 4, 9, and 17, of which clusters 9 and 17 were NKT cell clusters (*Nkg7* ^+^). T‐cell clusters 3 and 4 were *Cd4* ^+^ T cells, whereas cluster 1 appeared to contain cytotoxic *Cd8* ^+^ T cells. Stromal cells accounted for approximately 16% of all cells in the scRNA‐seq data. Clusters 5, 8, and 15 contained fibroblasts (*Cola1* ^+^ *Cola2* ^+^), whereas cluster 7 contained cells that are likely to be smooth muscle cells (*Tagln* ^+^ *Acta2* ^+^). Epithelial cells accounted for 8% of the total cell population (keratin‐expressing cells, including *Krt5* ^+^, *Krt6a* ^+^, and *Krt18* ^+^), composed of clusters 10 and 6. Clusters 13 and 19 were myeloid cells composed of macrophages/monocytes (*Cd68* ^+^ *Itgam* ^+^) and dendritic cells (DCs) (*Flt3* ^+^) (Figure 3B; supplementary material, Table S3); these cells made up 4% of the cells. A small population of endothelial cells (*Pecam1* ^+^), making up 0.4% of the total, was identified in cluster 21. Cellular composition and proportions were similar between prostates harvested 21 and 35 days after the booster, with few differentially expressed genes (Figure 3C,D; supplementary material, Figure S5B and Tables S5 and S6).

Comparison of the C57BL/6^*Aire*−/−^ scRNA‐seq data with human BPH tissue [10, 17] (Figure 3E–G) showed considerable similarities and some notable differences. In both C57BL/6^*Aire*−/−^ and human BPH samples, nonimmune cells were dominated by stromal and epithelial cells, with a minor endothelial population of cells present. In C57BL/6^*Aire*−/−^ mouse, the leukocyte (*Cd45* ^+^) fraction of cells was dominated by lymphocytes, whereas in the human BPH samples, lymphocytes and myeloid‐cell populations comprised a nearly equivalent proportion of the total immune cells. The prevalence of T cells, B cells, and myeloid cells observed in these data is reminiscent of that seen in the prostates of men diagnosed with BPH [10, 48, 49], though a much greater proportion of B cells was identified in the mouse prostates than in the human BPH specimens (Figure 3G). Conversely, a much greater proportion of myeloid cells was identified in human BPH specimens than in the C57BL/6^*Aire*−/−^ mouse, both in histology and scRNA‐seq data. In the scRNA‐seq data, epithelial cells were far more prevalent in the human data than in the C57BL/6^*Aire*−/−^, though this difference was not reflected in the histology. Another notable difference was that mast cells constituted a distinct population of immune cells in the human BPH samples (Figure 3F), but in the C57BL/6^*Aire*−/−^ samples, mast cells (*Kit*) did not form a distinct cluster (supplementary material, Figure S5C). Furthermore, in BPH patients, 3.73% of the total cells were mast cells, whereas fewer mast cells were observed in the prostates of both C57BL/6^*Aire*−/−^ (1.11% of total cells) and of WT C57BL/J6 mice (1.01% of total cells) [48, 50], which were used as a control.

### T‐cell subclustering indicates the presence of *Gzmk* ^hi^ T‐cell subpopulations in C57BL/6^*Aire*^ ^−/−^ prostates

**Figure 4:**
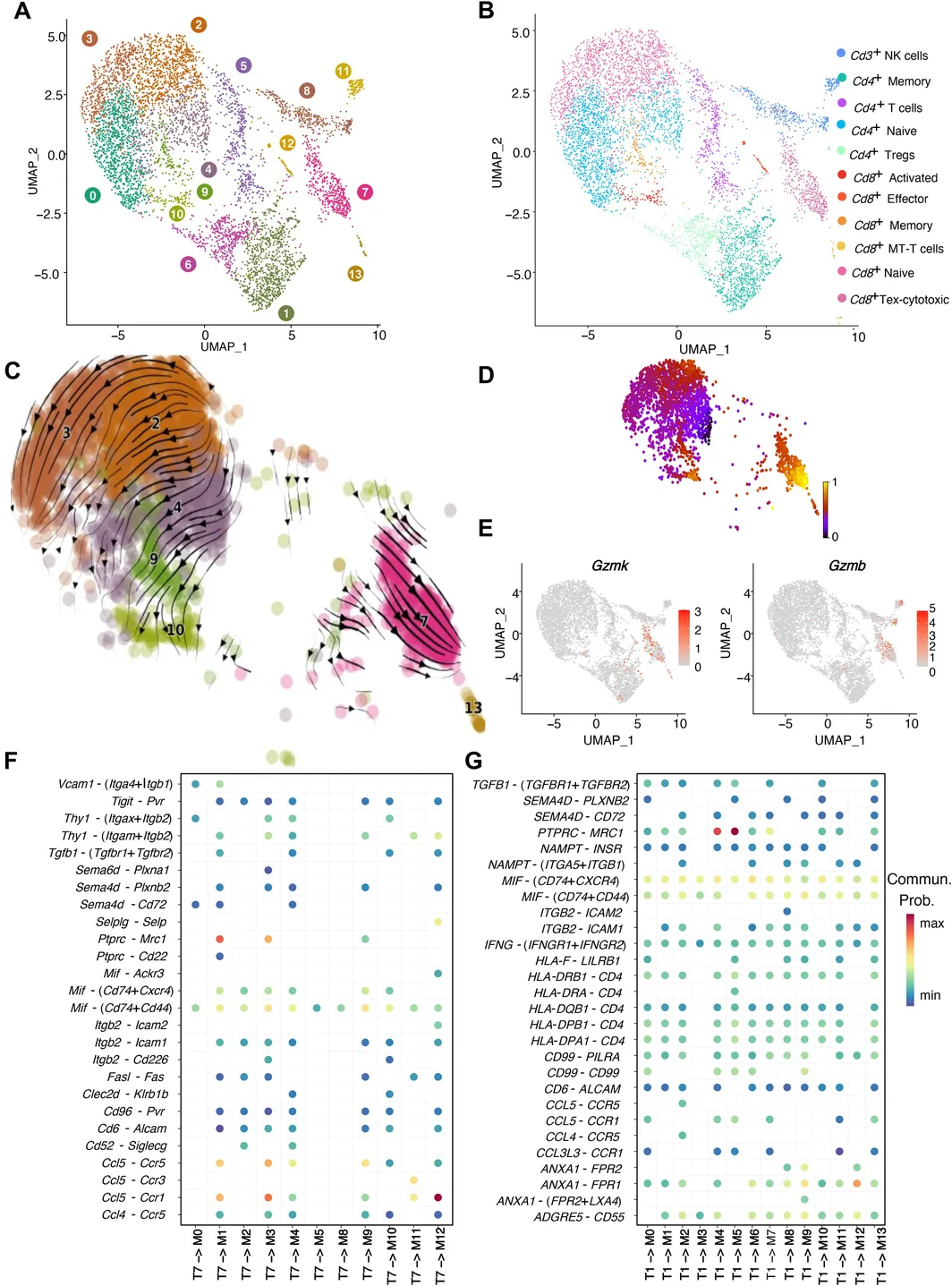
C57BL/6^*Aire*−/−^ prostate T‐cell subclustering. (A) UMAP of C57BL/6^*Aire*−/−^ prostate T‐cell subclustering. (B) ProjectTILS [51] annotated UMAP of C57BL/6^*Aire*−/−^ prostate T‐cell subclustering. (C) Embedding stream diagram of *Cd8* ^+^ T cells. (D) *Cd8* ^+^ T cells colored by estimated latent time. (E) Feature plots highlighting cluster 7 as mix of *Cd8* ^+^ *Gzmk* ^+^ *Tox* ^+^ cluster of T cells that is *Gzmb* ^−^ and others that are *Gzmb* ^+^. (F) Predicted interactions between aging‐associated T cells (T cell cluster 7) and myeloid‐cell clusters from C57BL/6^*Aire*−/−^ prostates. All interactions shown are statistically significant (adjusted *p* value <0.01), and points are colored according to the probability of a communication event occurring. The *x*‐axis denotes the clusters that are interacting with T cell clusters expressing the ligands and myeloid‐cell clusters expressing the receptors. The *y*‐axis denotes the ligands and receptors involved in the predicted interaction events. (G) Predicted interactions between aging‐associated T cells (T cell cluster 1) and myeloid‐cell clusters from prostates of BPH patients [10, 17]. All interactions shown are statistically significant (adjusted *p* value < 0.01), and points are colored according to the probability of a communication event occurring. The *x*‐axis denotes the clusters that are interacting with T‐cell clusters expressing the ligands and myeloid‐cell clusters expressing the receptors. The *y*‐axis denotes the ligands and receptors involved in the predicted interaction events.

Subclustering of CD3^+^ T cells and CD3^+^ NK cells led to the identification of 14 distinct subclusters (Figure 4A). A comparison with young, WT C57BL/6J mice [48, 50], used as controls, found few T cells in the prostate (only 76 total T lymphocytes were identified across both samples; data not shown). The identification of subclusters was both empirically determined using cluster markers (supplementary material, Figure S6A and Table S7) and established T‐cell subtype markers (supplementary material, Figure S6B) and programmatically determined using ProjectTILs [51] (Figure 4B; supplementary material, Table S8). Many T‐cell populations showed signatures similar to precursor‐exhausted T cells (*Tpex*). RNA velocity analysis placed cluster 2 *Cd8* ^+^ naïve T cells at the initial state and cluster 7 cells at the terminal state (Figure 4C,D). Cluster 7 appeared to contain a mix of *Gzmb* ^hi^ and *Gzmb* ^low^ cells, some of which were a *Gzmk* ^hi^ *Gzmb* ^low^ *Tox* ^hi^ subset of *Cd8* ^+^ cytotoxic T cells (Figure 4E) that have a gene expression profile similar to that of exhausted T cells (Figure 4B). The RNA velocity showed that the most terminal population of these cells was *Gzmb* ^high^, which fits with previous observations regarding granzyme expression and cytotoxic T‐cell differentiation in humans, specifically that few *CD8* ^+^ T cells express both *GZMK* and *GZMB* and that cells in earlier stages of differentiation tend to be *GZMK* ^high^ *GZMB* ^low^, and at later stages of differentiation they shift to *GZMK* ^low^ *GZMB* ^high^ [52, 53]. Interestingly, these *Gzmk* ^hi^ *Gzmb* ^low^ cells appeared to be analogous to aging‐associated T cells (Taa), a subset of *CD8* ^+^ T cells described previously in human BPH tissues [49, 54] (Figure 4E; supplementary material, Figure S6D).

An interaction analysis was performed to predict which myeloid cells the Taa cells would interact with (Figure 4F). Cluster 7 Taa cells interacted with all four macrophage clusters (0, 1, 3, and 12) and specifically interacted only with those that showed an M1‐like or mixed M1‐ and M2‐like phenotype. Notably, the interaction analysis between Taa and myeloid cells mirrored those seen in human BPH, in that Taa cells also largely interacted with myeloid cells along similar axes (Figure 4G). Although IHC confirmed the close spatial proximity of macrophages and T cells, supporting the plausibility of these interactions, it should be noted that the findings were inferred from scRNA‐seq data and therefore warrant further validation (supplementary material, Figure S7).

### Macrophage subclusters express a spectrum of M1 through M2 polarization signatures in C57BL/6^*Aire*^ ^−/−^ mouse prostates

**Figure 5:**
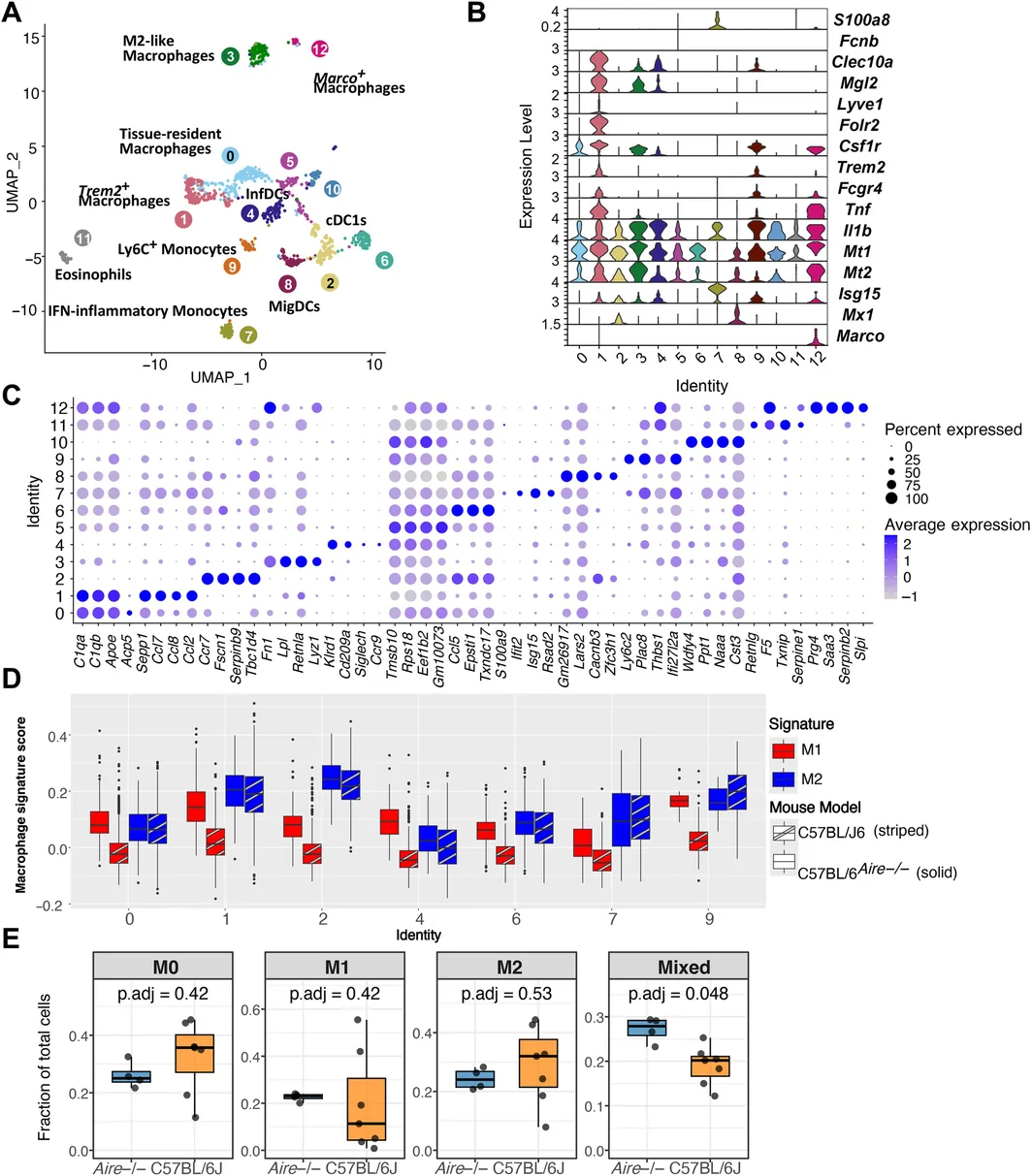
C57BL/6^*Aire*−/−^ prostate myeloid‐cell subclustering. (A) UMAP of myeloid‐cell subclustering. A total of 13 distinct clusters were identified and are highlighted. (B) Stacked violin plot of genes used to aid in myeloid‐cell identification. (C) Dot plot showing statistically significant marker genes (FDR < 0.01) of each myeloid subcluster. (D) Boxplots of macrophage signature module scores to determine whether an M1‐ or M2‐like gene expression program is expressed by various macrophage populations sequenced from both WT C57BL/J6 [48] and C57BL/6^*Aire*−/−^ prostates. (E) Proportions of macrophages displaying M0, M1, M2, or mixed polarization in C57BL/6^*Aire*−/−^ and C57BL/6J mouse prostates, with adjusted *p* values denoting significant differences.

Myeloid cells are known to increase in numbers in human BPH as prostate size increases, and macrophages have been found to be a highly abundant immune cell population in BPH tissues, second only in number to T cells [10]; hence, we performed a subclustering of myeloid cells to investigate the myeloid composition of C57BL/6^*Aire*−/−^ mice. Significantly fewer myeloid cells were observed in the C57BL/6^*Aire*−/−^ mice compared to in human BPH prostates (Figure 3G, adjusted *p* value = 0.028). Overall, 13 clusters of myeloid cells were identified (Figure 5A). SingleR [47], along with marker genes, was used to identify clusters of cells (Figure 5B,C; supplementary material, Figure S8A,B and Table S9). Clusters 0, 1, 3, and 12 were identified as macrophages (442 cells), clusters 2, 4, 5, 6, 8, and 10 as DCs (473 cells), clusters 7 and 9 as monocytes (117 cells), and cluster 11 as eosinophils (45 cells).

The macrophage clusters included two likely tissue‐resident macrophages (clusters 0 and 1), evidenced by *C1qa*, *C1qb*, and *Apoe* expression [55] and supported by yolk‐sac‐derived gene signature analysis [56] (supplementary material, Figure S8C and Table S10). Only cluster 1 expressed high *Trem2*, identifying it as lipid‐associated tissue‐resident macrophages implicated in human BPH [17, 25, 57]. Cluster 3 was identified as alternatively activated (M2) macrophages due to the expression of *Retnla* (Figure 5C) [58]. Cluster 12 appeared to be a population of *Marco* ^+^ macrophages (Figure 5B), and both clusters 1 and 12 exhibited inflammatory properties (*Tnf* expression) (Figure 5B). The abundance of *TREM2* ^+^ macrophages and *MARCO* ^+^ macrophages was positively correlated with BPH patient metrics and were previously reported in human BPH tissues using scRNA‐seq, histology, and cell flow cytometry [17]. Both clusters 3 and 12 also highly expressed *S100a4*, encoding a calcium‐binding enzyme, and similar macrophages increased pulmonary fibrosis through the activation and proliferation of lung fibroblasts [59, 60].

Among the monocyte clusters, based on the high expression of *Isg15*, *IL1B*, and *S100A8*, it appeared that cluster 7 was composed of interferon‐inflammatory monocytes (Figure 5B,C), which are often found in severe viral infections and autoimmune diseases [61, 62, 63]. Cluster 9 (*Ly6c2*, *Thbs1*) appeared to be a cluster of classical inflammatory monocytes (*Ly6C* ^+^) [64]. Among the DCs, clusters 10, 2, and 6 (*Xcr1*, *Batf3*, *Irf8*) appeared to be conventional type 1 DCs (cDC1s) [65, 66]. Cluster 8 (*Cacnb3*) was identified as migratory dendritic cells (migDCs) [67]. DC clusters 4 and 5 (*S100a8*, *Is1b*, *Cd83*) are likely to be inflammatory DCs (infDCs), which have been found to differentiate from monocytes that are recruited to sites of tissue damage and inflammation [68]. Unlike in human BPH prostates [17], no cluster of conventional type 2 DCs was observed.

A signature analysis of macrophage subpopulations with M1 and M2 gene expression was performed. This analysis determined that the largest macrophage cluster, cluster 0, had a primarily M1‐like phenotype and that cluster 3, not surprisingly given the high expression of *Retnla*, had an M2‐like phenotype, but the other two clusters (1 and 12) appeared to have a mixed phenotype (supplementary material, Figure S8D). The presence of M2‐polarized macrophages marks a distinction from human BPH, in that previously, no M2‐polarized macrophages were identified in the prostates of BPH patients [17]. However, the prevalence of mixed macrophages in C57BL/6^*Aire*−/−^ prostates aligns with the presence of *TREM2* ^+^ and *MARCO* ^+^ macrophages in human BPH, which are known to increase significantly in patients with the disease [17].

An integrative analysis of the C57BL/6^*Aire*−/−^ myeloid cells with those of normal WT C67BL/J6 mice [48] was performed and did not identify any clusters of myeloid cells that appeared to be unique to the C57BL/6^*Aire*−/−^ mouse prostate, though the inflammatory DCs in cluster 10 of the integrated clustering were far more prevalent in the C57BL/6^*Aire*−/−^ mouse prostates (supplementary material, Figure S8E,F). However, the C57BL/6^*Aire*−/−^ had a much higher number of macrophages that appeared to have a mixed M1‐ and M2‐like phenotype (Figure 5D,E). The presence of M1‐like, M2‐like, and mixed phenotypes mirrored what has been seen in macrophage populations derived from the prostates of patients diagnosed with BPH [17].

### Fibroblasts show the presence of senescence‐associated secretory phenotype and potential senescence signatures in C57BL/6^*Aire*^ ^−/−^ mouse prostates

**Figure 6:**
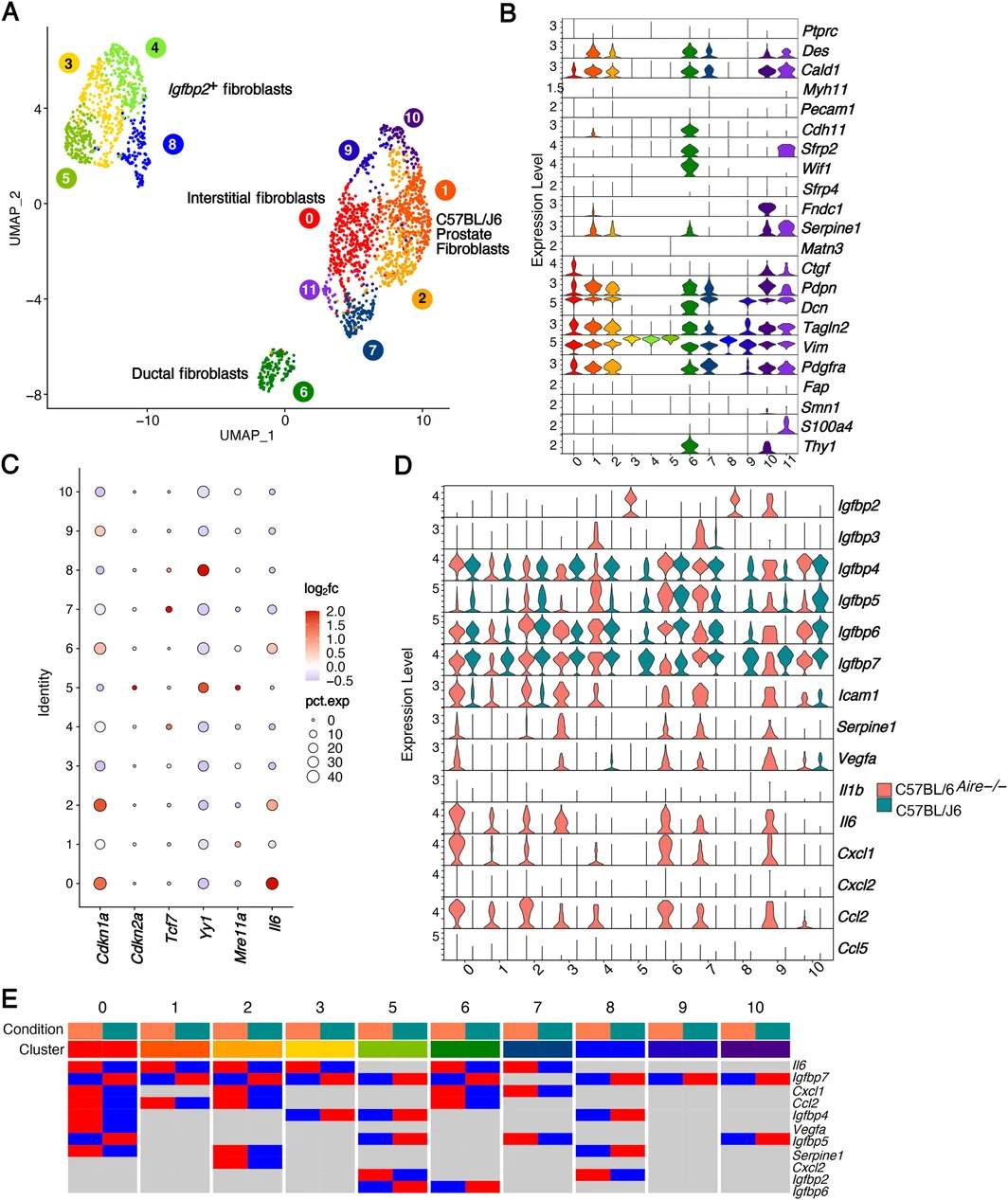
C57BL/6^*Aire*−/−^ prostate fibroblast subclustering. (A) Subclustering of fibroblasts. (B) Stacked violin plot of marker genes. Gene expression of these markers indicates that these cells are fibroblasts. (C) Senescence‐associated gene expression among fibroblast subclusters in C57BL/6^*Aire*−/−^ mice and in WT C57BL/J6 mice [48], which were combined and integrated. Dots are colored by log_2_ fold‐change of gene expression in C57BL/6^*Aire*−/−^ mice compared to WT C57BL/J6 mice [48]. (D) Split stacked violin plot of genes associated with a senescence‐associated secretory phenotype (SASP) in C57BL/6^*Aire*−/−^ mice and in WT C57BL/J6 mice [48]. (E) Binary heatmap highlighting SASP‐associated genes in each fibroblast subcluster that are differentially expressed between the prostates of C57BL/6^*Aire*−/−^ mice compared to in WT C57BL/J6 mice, with FDR controlled at 1%.

Due to the potential that GZMK‐expressing Taa cells could contribute to nonresolving inflammation through communication with fibroblasts [49], a subclustering of all fibroblasts, excluding smooth muscle cells (*Acta2*), was performed (Figure 6A) in an attempt to reveal heterogeneity in stromal cells. Fibroblast identity was confirmed for each cluster by both SingleR and marker expression (Figure 6B). Fibroblasts formed 12 subclusters in total, and markers were identified (supplementary material, Table S11). Fibroblast subclusters were compared with published datasets of fibroblasts from the prostates of young, healthy WT mice and human donors [48], as well as with those from the prostates of BPH patients [48]. Classification of fibroblast subclusters with the gene expression signatures derived from these publicly available datasets using Garnett identified most cells from cluster 0 (Figure 6A, supplementary material, Figure S9A–D and Table S12) as being highly similar to human interstitial fibroblasts, such as those identified previously from healthy prostates [48]. Like human interstitial fibroblasts, *Gpx3* and *Apod* were statistically significant [false discovery rate (FDR) < 0.01] markers of cluster 0 (supplementary material, Table S11). Clusters 1, 2, 7, and 10 were identified as similar to the prostate‐derived fibroblasts from WT mice (Figure 6A; supplementary material, Figure S9A and Table S12), and in total, 91%, 76%, 71%, and 67% of the cells in clusters 1,2, 7, and 10 were classified as WT mouse prostate fibroblasts, respectively (**s**upplementary material, Table S12). Like the WT prostate fibroblasts [48], clusters 1, 2, and 7 expressed high levels of the genes *C3*, *Ebf1*, *Gpx3*, *Sult1e1*, and *Igf1* (supplementary material, Table S11), which were identified as statistically significant (FDR < 0.01) markers of these clusters. Cluster 10 also expressed high levels of these genes. However, *Gpx3* and *Sult1e1* were not statistically significant markers of cluster 10. Clusters 3, 4, 5, and 8 had gene expression profiles similar to two fibroblast populations that were identified from the prostates of men with BPH [48]. They expressed high levels of *Igfbp2*, a component of the senescence‐associated secretory phenotype (SASP) seen in a number of aging‐related diseases [69] and also found at high levels in aged fibroblasts [70, 71]. Cluster 6 appeared to be the ‘ductal fibroblasts’ described in WT mouse prostates (Figure 6A; supplementary material, Figure S9A and Table S12) [48]. The cells in cluster 6 likewise expressed high levels of *Wnt2*, *Rorb*, *Wif1*, *Ifitm1*, and *Srd5a2* (supplementary material, Figure S9B and Table S11), which were previously identified as markers of ductal fibroblasts in WT mice and were statistically significant markers of cluster 6 (FDR < 0.01). Clusters 9 and 11 were also annotated ductal fibroblasts classified as ductal (supplementary material, Figure S9A and Table S12); however, with the exception of Cluster 11 expressing moderate levels of *Ifitm1*, neither cluster expressed high levels of ductal fibroblast markers (supplementary material, Figure S9B and Table S11), and none of the ductal fibroblast markers were statistically significant markers of these clusters.

Fibroblasts derived from the prostates of men diagnosed with BPH also showed expression of numerous markers of cellular senescence, specifically of the genes *CDKN1A*, *CDKN2A*, *TCF7*, *ME11A*, *YY1*, and *IL6* [49]. Likewise, a recent study found that fibroblasts from human BPH patients also expressed high levels of genes associated with a SASP [49]. We next integrated scRNA‐seq data from the prostates C57BL/6^*Aire*−/−^ and normal WT C67BL/J6 mice (supplementary material, Figure S9E,F) to determine whether there was a difference in the expression of SASP genes. This integrated analysis showed that, indeed, the majority of senescence markers showed higher expression in C57BL/6^*Aire*−/−^ than in normal WT C67BL/J6 mice (Figure 6C). Likewise, all C57BL/6^*Aire*−/−^ fibroblast clusters expressed some markers of SASP (Figure 6D,E), and, unlike in the WT C67BL/J6 mice, the majority of clusters in C57BL/6^*Aire*−/−^ (0,1, 2, 3, 4, 6, 7, and 9), particularly cluster 0, expressed particularly high levels of such markers.

## Discussion

The complex interplay of factors in BPH complicates the selection of research models. We characterized the C57BL/6^*Aire*−/−^ inflammation‐driven prostatic hyperplasia model, a benign autoimmune model that displays human BPH characteristics, including persistent inflammatory macrophage profiles with M1‐like macrophages and clearly defined neither M1 nor M2 phenotypes [54]. *Trem2* ^+^ and *Marco* ^+^ macrophages are present, analogous to T + E2 models [22] and human BPH prostates, where prevalence correlates with symptom severity. C57BL/6^*Aire*−/−^ prostates exhibit inflammaging markers (SASP, senescence markers in fibroblasts) and *Gzmk* ^hi^ *Gzmb* ^low^ *Cd8* ^+^ T cells seen in human BPH [45]. Taa cells accumulate with age, contributing to inflammation through Gzmk secretion, making this model useful for studying BPH immune/stromal dysregulation and prostatic inflammaging effects.

Notable differences exist between human BPH prostates and C57BL/6^*Aire*−/−^ mice, some likely species‐specific, others potentially methodological. Mast cells, abundant in the human BPH transition zone and increasing with prostate volume, made up lower proportions in both C57BL/6^*Aire*−/−^ and WT mice, a species‐specific limitation for mast cell research utility. Human samples had higher myeloid‐cell fractions than mouse samples, possibly due to increased heterogeneity or differences in digestion protocols. Some differences noted in the scRNA‐seq data appear methodologically skewed: while lymphocytes are abundant in both species, human BPH showed more T than B cells in both scRNA‐seq and histology, whereas C57BL/6^*Aire*−/−^ mice showed more T than B cells in histology, but the reverse in scRNA‐seq.

All mouse models for BPH‐related urinary symptoms have limitations, yet preclinical models are critical for dissecting the complex mechanisms driving epithelial and stromal hyperplasia. Future work will compare the C57BL/6^*Aire*−/−^ model with other BPH models, analyzing immune, epithelial, and stromal cell composition alongside functional outcomes like voiding behavior. Based on this study, the C57BL/6^*Aire*−/−^ model is highly relevant for BPH research, particularly for studying the impact of chronic, nonresolving prostatic inflammation seen in patients, as it accurately recapitulates the immune cell composition and epithelial‐stromal expansion observed in human disease. Given clinical evidence that TNF inhibition reduces prostate size [10], this model is a valuable system for evaluating anti‐TNF and other anti‐inflammatory therapies. Furthermore, the model can be used to investigate age‐related inflammatory processes and the role of myeloid‐cell dysfunction in prostate pathology.

## Author contributions statement

TLR and SWH created the study concept. NAL, TLR and SWH designed research studies. MMB, DW, JY, APG, BTH, SWH and REV were involved in the acquisition of tissues. NAL, HK and AKK performed statistical and bioinformatics analyses. NAL, MMB, HK, AKK, JY, DW and REV performed data acquisition, and all authors contributed to data analysis and interpretation. NAL, MMB, HK, TLR, SWH and REV wrote the manuscript, and all authors critically revised the manuscript.

## Supporting information


**Figure S1**. C57BL/6^*Air*e−/−^ stromal lymphoid aggregates
**Figure S2**. Cell flow cytometry shows inflammatory cell distribution in different prostate lobes of C57Bl/6^*Aire*−/−^ and control mice (C57Bl/6^*Aire*+/+^ and C57Bl/6^*Aire*+/−^)
**Figure S3**. Representative H&E image of seminal vesicle of C57Bl/6^*Aire*−/−^ mouse lacking significant immune cell infiltration
**Figure S4**. Urinary tract function in C57BL/6^*Aire*−/−^ and control WT C57BL/6^*Aire+*/+^ mice as analyzed by void spot assay (VSA)
**Figure S5**. Combined clustering of cells from C57BL/6^*Aire*−/−^ mouse prostates
**Figure S6**. T‐cell subclustering
**Figure S7**. CD11b IHC of C57BL/6^*Aire*−/−^ mouse prostates
**Figure S8**. Myeloid‐cell subclustering
**Figure S9**. Fibroblast‐cell subclustering

**Table S1.** Flow antibody clones.
**Table S2**. Run metrics for scRNA‐seq of cells from C57BL/6^*Aire*−/−^ mouse prostates
**Table S3**. Clusters identified in unsupervised clustering analysis of scRNA‐seq data from C57BL/6^*Aire*−/−^ mouse prostates
**Table S4**. Marker genes for combined clustering of all cell types identified in C57BL/6^*Aire*−/−^ mouse prostates
**Table S5**. Pseudobulk differentially expressed genes identified between time points
**Table S6**. Clusterwise differentially expressed genes identified between time points
**Table S7**. T‐cell subclustering markers
**Table S8**. T‐cell subclustering identities determined by projectTILS, SingleR using ImmGen database, and final determination made through marker identification in conjunction with automatic assignment
**Table S9**. Myeloid‐cell subclustering markers
**Table S10**. Yolk‐sac‐derived macrophage percentages per cluster
**Table S11**. Fibroblast subclustering markers
**Table S12**. Fibroblast annotation

## Data Availability

The scRNA‐seq data described in this manuscript are available in Gene Expression Omnibus under accession number GSE300554. Additional requests for further information about these studies should be directed to the corresponding author and will be delivered upon reasonable request.
