# Supplementary material for: Immune dysregulation in the prostates of C57BL/6 Aire−/− mice mirrors that seen in human benign prostatic hyperplasia
Source: J Pathol. 2026 Jul 8;270(2):211–25. doi: 10.1002/path.70093 (PMC13576882; doi:10.1002/path.70093)
Supplement: Supplementary file 1 [file PATH-270-211-s001.docx]

**Immune dysregulation in the prostates of C57BL/6*^Aire^*^−/−^ mice mirrors that seen in human benign prostatic hyperplasia**

NA Lanman *et al. J Pathol* <https://doi.org/10.1002/path.70093>

**Supplementary materials and methods**

**Supplementary Figures S1–S9**

**Supplementary Tables S1–S12 are provided in a separate Excel file**

Reference numbers refer to the main text list

**Supplementary materials and methods**

**Ethics approval**

All human tissue procurement was performed in accordance with protocols approved by the Endeavor Health (formerly NorthShore University HealthSystem) Institutional Review Board.

All mouse procedures were performed in accordance with Purdue Animal Care and Use Committee-approved protocols.

**Mice**

B6.129S2-*Aire^tm1.1Doi^*/J [RRID:IMSR_JAX:004743, The Jackson Laboratory (JAX), Bar Harbor, ME, USA] were backcrossed with C57Bl/6 mice for at least nine generations at JAX. C57Bl/6*^Aire+/−^* mice were purchased and bred to generate C57Bl/6*^Aire−/−^*, C57Bl/6*^Aire+/−^*, and C57Bl/6*^Aire+/+^* offspring. All mice were housed and maintained under pathogen-free conditions with 12-h light/12-h dark cycles.

To collect prostate homogenate protein, prostates from 8- to 12-week-old male C57BL/6 mice were collected, minced, digested in complete RPMI + 1 mg/ml collagenase for 1 h, then heat shocked at 95 °C for 5 min. Protein was quantified on a NanoDrop Spectophotometer (DeNovix Inc, Wilmington, DE, USA) and stored at −20 °C.

**Mouse necropsy and histology**

Samples of mouse prostate as well as lung, kidney, liver, spleen, and pancreas were collected into 10% NBF, embedded in paraffin, and sectioned and stained with H&E. Extraprostatic tissues were within normal gross and histological limits, meaning no significant changes were noted in these tissues. Prostate tissues were also sectioned and stained with toluidine blue histochemical stain. For prostate IF or IHC staining, FFPE sections were deparaffinized and antigen retrieval was performed at 95 °C for 20 min in a BioCare decloaking chamber (BioCare Medical, Pacheco, CA, USA). Sections were stained with CD8a (1:800, clone EPR20305, Abcam, Cambridge, UK) and Cd4 (1:200, clone 4SM95, Invitrogen, Waltham, MA, USA) primary antibodies (IF) or Cd20 (1:100, clone EP459Y, Abcam) primary antibody (IHC) in a BioCare intelliPATH stainer (BioCare Medical) at room temperature (RT). Slides were either digitized on a Leica Versa8 whole-slide scanner (Leica Biosystems, Wetzlar, Germany) or imaged with an Olympus BX51 microscope (Evident Scientific, Inc., Tokyo, Japan) and an Olympus DP80 camera (Evident Scientific, Inc.). For IHC quantification (positive cells per mm^2^), C57Bl/6*^Aire−^*^/−^ and C57Bl/6*^Aire^*^+/−^ scanned slides were analyzed using Qupath version 5.0.1 software [85] Prostate mast cells were quantified (mast cells per mm^2^) by counting toluidine blue-staining mast cells within three C57Bl/6*^Aire−/−^* and three C57Bl/6*^Aire^*^+/+^ prostate tissues in 20 high-powered (400×) fields, then dividing by the total examined area (4.74 mm^2^).

**Void spot assay**

Mice were separated (one mouse/cage) into cages lined by Whatman 3MM Chr chromatography paper (Cytiva/Whatman, Marlborough, MA, USA) without food or water for 3 h (3–6 p.m.). Images of urine-stained papers were taken under UV light using ChemiDoc Imaging System (Bio-Rad, Hercules, CA, USA) and analyzed using the Void Whizzard software plugin to ImageJ (National Institutes of Health, Bethesda, MD, USA).

**Human prostate specimens**

Human prostatic tissues were obtained as previously described [10,17]. Specimens were fixed in 10% NBF, embedded in paraffin, and sectioned and stained with H&E. For IHC, FFPE sections were deparaffinized, and antigen retrieval was performed at 95 °C for 20 min in a BioCare decloaking chamber (BioCare Medical). Slides were cooled for 20 min at RT and transferred to TRIS buffer with Tween 20 detergent (TBST) then incubated with 3% hydrogen peroxide in water for 5 min. Slides were rinsed with TBST and incubated in 2.5% normal goat serum for 20 min. Sections were stained using primary antibodies against CD8a (clone C8/144B, Abcam), CD4 (clone EPR6855, Abcam), or CD79a (clone SP18, Invitrogen) in a BioCare Intellipath stainer (BioCare Medical) at RT. Slides were rinsed twice in TBST, and a goat anti-rabbit secondary (MP-7451, Vector Labs, Newark, CA, USA) (CD4, CD79a) or horse anti-mouse secondary (MP-7402, Vector Labs) (CD8a) was applied for 30 min. Slides were rinsed twice in TBST, and Vector ImmPACT DAB (SK-4105, Vector Labs) was applied for 5 min. Slides were rinsed in water and transferred to a Leica Autostainer XL for hematoxylin counterstain, dehydration, and coverslipping. Stained slides were digitized on a Leica Versa8 whole-slide scanner (Leica).

**Cell flow cytometry**

All four lobes of mouse prostates were collected from C57BL/6*^Aire−/−^* mice, digested, and processed to a single-cell suspension followed by cell flow cytometry, as previously described [30,38]. Prostates were minced, digested in 1 mg/ml collagenase (Sigma-Aldrich, St. Louis, MO, USA) in RPMI-1640 (Gibco, Waltham, MA, USA) medium containing 10% FBS (Corning, Corning, NY, USA) with shaking at 37 °C for 2 h, then filtered through a 70-μm-mesh filter. Cells were suspended in 100 μl complete RPMI with 2 μl mouse TruStain FcX antibody (Biolegend, San Diego, CA, USA) and 1 μl Zombie Violet Fixable Viability dye (Biolegend) per sample and incubated at RT for 10 min in the dark. Cells were then divided and incubated with cocktails of cell surface antibodies (Biolegend) listed below at 1 μl in 100 μl PBS at 4 °C for 30 min in the dark. Cells were rinsed and fixed in 10% NBF for 10 min. Samples were run on a BD Fortessa Flow Cytometer (BD Biosciences, Franklin Lakes, NJ, USA) and analyzed with FlowJo software (BD Biosciences).

The flow antibody clones that were used are provided in the supplementary material, Table S1. Myeloid markers: Cd45 (clone 30-F11), Cd11b (clone M1/70), Gr-1 (RB6-8C5), F4/80 (clone BM8); Lymphoid marker: Cd45 (clone 30-F11), Cd3ε (clone 500A2), Cd8a (clone 53-6.7), B220 (clone RA3-6B2), Foxp3 (clone MF-14); aging-associate T cell (Taa): Cd45 (clone 30-F11), Cd8a (clone 53-6.7), Pd-1 (clone 29F.1A12), Tox (clone TXRX10).

**Single-cell RNA sequencing**

Six male C57BL/6A*^Aire−/−^* mice were used in scRNA-seq experiments, with prostates pooled from three mice for each scRNA-seq sample. Half of all four prostate lobes of prostates were extracted, pooled, and then digested in complete RPMI with 1 mg/ml Collagenase I on a shaker at 225 rpm and 37 °C for 1.5 h. Samples were filtered through a 70-μm strainer, centrifuged at 250 × *g* for 5 min, and resuspended. Dead cells were removed using the EasySep Dead Cell (Annexin V) Removal Kit (STEMCELL Technologies, Vancouver, BC, Canada) following the manufacturer’s protocol. Cells were counted with trypan blue and diluted to a concentration of 1,000 cells/μl, prepped with the Chromium Next GEM Single Cell 3' Kit version 3.1 (10x Genomics, Pleasanton, CA, USA) following the manufacturer’s protocol, and two wells per sample were loaded into the 10x Chromium (10x Genomics) instrument following the manufacturer’s protocol for a recovery target of 10,000 cells. RNA clean-up and library preparation were performed following the 10x Genomics protocols.

**ScRNA-seq analysis of C57BL/6*^Aire−/−^* data**

Quality control and alignment of the scRNA-seq datasets to the GRCm38 reference genome was performed using CellRanger version 2.1.0 (10x Genomics). Matrices were imported into R version 4.1.1 and analyzed using Seurat version 4.1.0 R [42,43]. Initial cell type identities were assigned using the SingleR version 1.4.0 algorithm [44] using the Immunological Genome Project reference. High-quality cells that had between 300 and 3,000 detected features and mitochondrial read content  < 22% were retained for further analysis. Hemoglobin genes, mitochondrial read count, and number of UMIs were regressed out computationally, and data were normalized using SCTransform [45]. The datasets were integrated and clustered using shared nearest-neighbor graph construction and Louvain-based community detection. Contaminant cell populations from seminal vesicles were identified using the *Svs* gene and removed before reintegration and reclustering on the top 17 principal components at a resolution of 0.6, with the optimal resolution identified using the clustree version 0.4.4 R package [46]. Differential expression analysis was performed using limma version 3.50.0 [47]. Markers were identified between the clusters by performing the Wilcoxon rank-sum test on the normalized RNA assay. Marker genes were defined as having log_2_ fold-change > 0.25 and adjusted *p* value < 0.01. Pseudobulk analysis between time points was done using negative binomial tests within Seurat by aggregating the cells based on the time points and samples using the AggregateExpression() function. Differentially expressed genes are defined as having adjusted *p* value < 0.05. Gene Ontology (GO) and pathway enrichment analysis on cluster marker genes was performed using the ClusterProfiler version 4.2.2 [48] and ReactomePA version 1.38.0 [49] with the org.Mm.eg.db version 3.14.0 mouse annotation [50]. RNA velocity analyses were performed using scVelo [51] version 0.2.4, and cell fate determination analyses were performed using CellRank version 1.5.1 [52].

**Subclustering analyses**

T cells, fibroblasts, and macrophages were further extracted for finer subclustering analyses. For the T-cell subclustering, we first selected cells from clusters 1, 3, 4, 9, and 17 and then added cells expressing the following markers: *Cd3d*, *Cd3g*, and *Cd3e*. Next, we removed cells expressing any of the following genes: *Col1a1*, *Col1a2*, *Acta1*, *Acta2*, *Cd68*, *Cd19*, *Ms4a1*, *Krt14*, *Krt19*, *Cdh1*, *Cd79a*, *Krt5*, *Krt8*, *Krt15*, *Krt18*, *Krt81*, *Krt8*. We further subclustered T cells into *Cd4^+^* and *Cd8^+^* cells by segregating cells expressing *Cd4* or *Cd8a*, respectively. Clusters were annotated using ProjectTILs with the associated mouse reference atlas [61]. For the fibroblast subclustering, we first selected cells from clusters 5, 7, 8, and 15 and then added cells expressing *Col1a1* or *Col1a2*. We then removed cells expressing any of the following genes: *Acta1*, *Acta2*, *Cd68*, *Cd19*, *Ms4a1*, *Krt14*, *Krt19*, *Cdh1*, *Cd79a*, *Krt5*, *Krt8*, *Krt15*, *Krt18*, *Krt81*, *Krt86*, *Cd3d*, *Cd3g*, or *Cd3e*. For the myeloid-cell subclustering we took cells from clusters 13 and 19 and added cells expressing *Cd68* to these cells and then removed cells expressing *Acta1*, *Acta2*, *Cd19*, *Ms4a1*, *Krt14*, *Krt19*, *Cdh1*, *Cd79a*, *Krt5*, *Krt8*, *Krt15*, *Krt18*, *Krt81*, *Krt86*, *Cd3d*, *Cd3g*, *Cd3e*, *Col1a1*, or *Col1a2*. We used a resolution of 1.0 and then removed a subcluster of cells (cluster 13) that was identified as fibroblasts by SingleR and expression of *Col1a1* or *Col1a2*. The subsets were then reclustered independently using graph-based clustering on principal components using a resolution of 0.4. In macrophage clusters, the analysis to quantify macrophage polarization was calculated using the addModuleScore() function in Seurat for M1 and M2 gene signatures. The feature sets used for M1 and M2 signatures were taken from a prior publication [62]. In the T-cell subclustering analysis, including both *Cd4*^+^ and *Cd8*^+^ T cells, 14 principal components and a resolution of 0.7 were used in clustering and community detection. A total of 11 principal components and a resolution of 0.8 was used in the fibroblast subclustering, whereas in the macrophage subclustering, 14 principal components and a resolution of 0.8 were used to cluster and identify communities. Differential expression between the new subclusters was determined using Wilcoxon rank-sum to identify subtype-specific markers. Cellchat version 1.6.1 was used to predict interactions between cells [53,72].

**Comparison of C57BL/6*^Aire−/−^* scRNA-seq with human BPH data and WT mice**

C57BL/6*^Aire−/−^* scRNA−seq data were compared to human BPH leukocyte scRNA-seq data from the transition zone of 10 small (<40 g) and 10 large (>90 g) prostates as well as scRNA-seq data from five large prostates from which all cell types were sequenced [10,17,54]. The C57BL/6*^Aire−/−^* scRNA-seq data were also compared with a previously published scRNA-seq dataset from the prostates of normal, WT C57BL/6J mice [55,56]. We mapped orthologous genes between humans and mice using g:Profiler's g:orth function. Genes flagged as “N/A” (indicating no mapping) were manually searched in NCBI and GeneCards to confirm the presence or absence of orthologs. Genes with multiple ortholog mappings were retained as in Do *et al* [57]. We used Garnett version 0.1.23 [58] to train a multinomial classifier based on the top 25 marker genes from fibroblast clusters in BPH, WT mice, and healthy human donors, considering 50 unknown cells as the outgroup. The FindAllMarkers function in Seurat version 4.1.3 [42,43] was used to identify genes in human BPH fibroblast clusters, selecting those with an adjusted *p* value of 0.01 and a positive fold-change using the Wilcoxon test. Marker genes for fibroblast clusters from healthy human donors and WT mice were obtained from a prior publication [55]. This trained classifier was then applied to identify cell types and extend assignments to derive cluster labels. To annotate the fibroblasts, the majority annotation was taken, provided that >50% of cells were annotated with the consensus annotation and that marker genes of those clusters also matched those of the reference population of cells. To perform supervised classification of myeloid subclustering to identify potential yolk-sac-derived macrophages, Garnett was used to train a human yolk sac classifier of the top 75 differentially expressed genes from a previous publication [59] using a minimum of eight representative cells and the default 500 unknown cells to be included as an outgroup.

Datasets from fibroblasts and myeloid/macrophages populations were obtained from the CELLxGENE browser for the WT C57BL/6J mice [55,56], and Ensembl-formatted gene identifiers were converted into gene symbols using the ensembl2sym function in scMiko 0.1 [60]. These were integrated with the respective subclusters from the C57BL/6*^Aire−/−^* data after performing a sctransform-based normalization regressing out differences in UMI and mitochondrial content in Seurat version 4.1.3 [42,43]. We used the first nine principal components and a resolution of 0.8 to integrate and identify clusters in the combined fibroblast population; for myeloid/macrophages 12 principal components at a resolution of 0.7 were used. The FindAllMarkers function from Seurat 4.1.3 [42,43] was used to identify the marker genes for each cluster in the two populations with an adjusted *p* value < 0.01 using the Wilcoxon test. The FindMarkers function employing a negative binomial test was performed to identify the differences between the WT mice and C57BL/6*^Aire−/−^* mice. Kyoto Encyclopedia of Genes and Genomes, Reactome pathways, and GO analyses on marker genes were performed using the ClusterProfiler version 4.2.2 [48] and ReactomePA version 1.38.0 [49] with the org.Mm.eg.db version 3.14.0 annotation package [50]. We used Garnett to identify M0, M1, M2, mixed, and yolk-sac-derived macrophages in the myeloid clustering and performed a signature analysis to determine M1 and M2 polarization as described earlier for the subclustering analyses. Differences in macrophage polarization between WT control C57BL/6J and C57BL/6*^Aire−/−^* mice were assessed using two-sided Wilcoxon rank-sum tests in R, with *p* values adjusted using the Benjamini–Hochberg method.

**Figure S1. C57BL/6*^Air^*^e−/−^ stromal lymphoid aggregates.** (A) Immunofluorescence highlighting nuclei (DAPI, left). CD4 (orange, center) or CD8 (green, right) cells. Scale bars, 100 μm. (B) Quantification of CD4^+^ and CD8+ T cells (per mm^2^) in dorsal, anterior, and ventral lobes in IHC sections. (C) Quantification of CD11^+^ myeloid cells (per mm^2^) in IHC sections. (D) Quantification of mast cells (per mm^2^) in toluidine blue-stained histological sections. Graphical data are represented as mean with standard deviation.

**Figure S2. Cell flow cytometry shows inflammatory cell distribution in different prostate lobes of C57Bl/6*^Aire^*^−/−^ and control mice (C57Bl/6*^Aire^*^+/+^ and C57Bl/6*^Aire^*^+/−^).** (A) CD45+ leukocytes are significantly increased in anterior and dorsal-lateral lobes of C57Bl/6*^Aire^*^−/−^ but not in ventral lobes. (B) Fraction of different leukocyte populations in different prostate lobes in C57Bl/6*^Aire^*^−/−^ and control mice. (C) Fraction of different T-cell populations in different prostate lobes in C57Bl/6*^Aire^*^−/−^ and control mice.

**Figure S3. Representative H&E image of seminal vesicle of a C57Bl/6*^Aire^*^−/−^ mouse lacking significant immune cell infiltration.** Note inflamed anterior prostate lobe (asterisk). Scale bar, 100 μm.

**Figure S4. Urinary tract function in C57BL/6*^Aire^*^−/−^ and control WT C57BL/6*^Aire+^*^/+^ mice as analyzed by void spot assay (VSA).** (A) Total number of void spots. (B) Total urine volume. (C) Urine volume/void spot.

**Figure S5. Combined clustering of cells from C57BL/6*^Aire^*^−/−^ mouse prostates.** (A) Cell identities as determined by SingleR. (B) UMAP of C57BL/6*^Aire^*^−/−^ mouse prostate-derived cells, colored by the number of days after the booster. (C) Feature plot showing *Kit* expression, highlighting mast cells.

**Figure S6. T-cell** **subclustering.** (A) Dotplot of top four statistically significant (FDR < 0.01) marker genes identified in each T-cell cluster. (B) Stacked violin plot of genes used to aid annotation of T-cell clusters. (C) ProjectTILS [51] annotated UMAP. (D) Cell flow cytometry of aging-associated T cells. PD-1^+^TOX^+^ cells were analyzed with flow cytometry of Day 31 post-immunization C57BL/6*^Aire^*^−/−^, C57BL/6*^Aire^*^+/−^, and C57BL/6*^Aire^*^+/+^ mouse prostates. Data are presented as mean ± SEM. There is no GZMK mouse antibody, so PD-1 and TOX antibodies were used to identify aging-associated T cells in mice, as was done in a previous study [54].

 **Figure S7. CD11b IHC of C57BL/6*^Aire^*^−/−^ mouse prostates.** CD11b^+^ myeloid cells (brown) are located in close proximity to and within organizing lymphoid structures. Scale bars, 100 μm.

**Figure S8. Myeloid-cell subclustering.** (A) Stacked violin plot of genes used in annotating myeloid-cell populations. (B) Cluster identities, as determined by the program SingleR. The majority of the cells present are macrophages and DCs. (C) Supervised classification identifies likely yolk-sac-derived macrophages [56] in the C57BL/6*^Aire^*^−/−^ myeloid populations, as shown in the UMAP by red points. (D) Boxplots of macrophage signature module scores of macrophage clusters from integrated C57BL/6*^Aire^*^−/−^ and normal WT C67BL/J6 [48] analysis to determine whether an M1- or M2-like gene expression program is expressed by various macrophage populations. (E) Integrated UMAP showing myeloid cells from C57BL/6*^Aire^*^−/−^ and WT C57BL/J6 mice [48]. (F) Split integrated UMAP showing combined integration of myeloid cells from C57BL/6*^Aire^*^−/−^ and WT C57BL/J6 mice [48].

**Figure S9. Fibroblast-cell subclustering.** (A) Fibroblast subclustering depicting cells classified using markers of prostate-derived fibroblasts from WT C57BL/6J mice [48]. (B) Stacked violin plot of ductal fibroblast marker expression in fibroblast subclustering. (C) Fibroblast subclustering depicting cells classified using markers of prostate-derived fibroblasts from healthy human prostates. (D) Fibroblast subclustering depicting cells classified using markers of prostate-derived fibroblasts from transition zone of men diagnosed with BPH. (E) UMAP of C57BL/6*^Aire^*^−/−^ stromal cells integrated with WT C57BL/J6 stromal cells [48]. (F) Split integrated UMAP showing fibroblasts from C57BL/6*^Aire^*^−/−^ and WT C57BL/J6 mice [48].
